# Notes and News

**Published:** 1898-08-27

**Authors:** 


### NOTES AND NEWS.

(Collected and Communicated.)

A fire occurred recently at the Quarrier Homes for Consumption. The conflagration broke out in the administration buildings, and fortunately did not spread any further. Twenty-two patients were in the wards at the time of the fire.

A curious point in poor-law regulations has just come prominently forward, owing to an unfortunate occurrence in the course of its enforcement. A labourer was taken to Guy's Hospital by his wife to be treated, as he appeared to be very ill. The doctor on duty regarding his case as being one of no extreme urgency, although he appeared very weak, gave him an order to go into the workhouse, and provided a cab to remove him, stating in his certificate that he regarded the man as a fit case for the infirmary. The patient died during the night, and the coroner naturally asked why he had not been sent to the infirmary, since his admission was suitable? The doctor then explained that the poor-law guardians will not accept patients into their infirmaries on certificates signed by the medical officers of hospitals alone; such certificates must be counter-signed by the district medical officer. As he considered that immediate admission was necessary, he sent him primarily to the workhouse. The coroner remarked that to regard a certificate signed by a hospital doctor as insufficient was absurd, and such a regulation should be altered. Of course, the intention of the guardians to safeguard themselves from the possibility of having patients undesired by the hospitals planted upon them is evident. But it would undoubtedly be better if a patient manifestly fitted in any doctor's opinion for an infirmary were sent primarily to it, as he might easily be removed to the workhouse if the infirmary medical officer did not concur with the verdict received from the hospital.

The Prince of Wales, whose condition is most satisfactory, has left Cowes in the Royal yacht for a short cruise.

An infirmary has been opened at Hebburn. Previously the sick poor had to be removed to the Newcastle Infirmary if they required hospital treatment. The new institution contains a large ward of 20 beds for male patients, a smaller ward of seven beds for women, an operating room, dispensary, and the usual offices.

After some consideration, a proposition to add an isolation ward to the Coventry and Warwickshire Hospital has been voted. Typhoid cases are received there in the general wards, and recently the City Hospital, which is rebuilding, made an application for permission to send on typhoid cases to the Coventry Hospital, but this has not been given owing to limited accommodation.

The recent sultry weather has been responsible for much illness, and many deaths. There are always a certain number of fatalities from heat, and atmospheric disturbances also claim their victims. It does not often happen, however, that thunder is attended with fatal effects. Nevertheless, such has been the case at Hammersmith, where a woman died of fright caused by a thunderstorm.

Belfast is visited by an alarming typhoid epidemic at the present time. During the present month upwards of 605 cases have occurred, and the last week shows a record of 267 fresh patients. Every ward in the fever hospital is full to overflowing, In the workhouse hospital over 300 cases are under treatment, and the nursing staff are quite broken down. The Poor Law Guardians have decided to secure the services of two additional doctors and of 12 nurses.

The sad case of a boy named Naylor, on whom an inquest was held on the 17th inst., brings once more into prominence the delays which sometimes occur in attending to patients in the receiving room of a large hospital like Guy's. The boy's injuries were very serious, as, according to the evidence, a stick, entering through the eye, had penetrated to the back of the orbit and injured the membrane covering the brain. When the parents brought the child to the hospital on the day of the accident the nature of the injury does not seem to have been apprehended, and the boy was sent home with a bandage over his eye, the parents being told to bring him back to the hospital on the following day. This case is one which shows the urgent difficulties under which casualty-room work is often carried on. It is clear from the full account of this case, given on page 378, that it was not an instance of careless oversight. The patient on his first visit was seen by the dresser, who drew the attention of the assistant house surgeon to the injury, and a careful examination was made. It is much to be regretted that the gravity of the accident was not suspected, but in such a case an accurate diagnosis of the condition may be absolutely impossible.

